# Relationship Between Sleep Irregularity and School Non-Attendance Among Japanese Elementary and Junior High School Students

**DOI:** 10.3390/children13010080

**Published:** 2026-01-04

**Authors:** Ikuko Hirata, Tomoko Nishimura, Yuko Osuka, Manabu Wakuta, Masako Taniike

**Affiliations:** 1 Department of Child Development, United Graduate School of Child Development, The University of Osaka, Osaka 5650871, Japan; nishimura.ugscd@osaka-u.ac.jp; 2 Institute of Child Developmental Science Research, Shizuoka 4300929, Japan; yuko.osuka@kohatsu.org (Y.O.); manabu.wakuta@kohatsu.org (M.W.); 3 Molecular Research Center for Children’s Mental Development, United Graduate School of Child Development, The University of Osaka, Osaka 5650871, Japan; masako@kokoro.med.osaka-u.ac.jp

**Keywords:** school non-attendance, sleep regularity, insufficient sleep, delayed sleep–wake rhythm disorder

## Abstract

**Highlights:**

**What are the main findings?**

- Irregular sleep patterns are associated with school non-attendance.
- Students exhibiting irregular sleep patterns are at a higher risk of experiencing difficulties in academics, teacher–student relationships, friendships, and family relationships.

**What are the implications of the main findings?**

- Irregular sleep patterns are a barometer indicating the need for student intervention.
- Promoting the regularity of students’ sleep patterns may possibly prevent school non-attendance.

**Abstract:**

**Background/Objectives**: In Japan, the number of elementary and junior high school students who do not attend school is increasing. Sleep problems are considered a contributing factor. **Methods**: This study utilized self-administered questionnaires about the sleep patterns and backgrounds of 25,257 students from the 3rd–10th grades across 91 elementary schools, 51 junior high schools, and 36 high schools in Japan. Latent class analysis was performed to assess sleep regularity. Logistic regression analysis was conducted to examine the relationship between sleep regularity and school attendance status, as well as the relationship with protective factors against non-attendance. **Results**: Overall, 19,005 students responded. The response rate was 75.2%. Sleep regularity was categorized into Class 1, Regular; Class 2, Somewhat Irregular; Class 3, Irregular; and Class 4, Schedule-Dependent. Class 1 decreased with grade, from 61.8% in the 3rd grade to 46.2% in the 10th grade. Class 3 comprised 10.0% of students not experiencing school non-attendance, 37.9% among students with persistent school non-attendance, and 17.9% among students who had resumed school attendance after school non-attendance in the previous year. Classes 2, 3, and 4 showed a negative relationship with protective factors against non-attendance such as good relationships with teachers and family, good communication, academic performance, proficiency in athletic activities, and the presence of a place to belong outside school. **Conclusions**: Sleep irregularity is related to school non-attendance and may serve as a barometer of students’ communication and academic difficulties. Additionally, we propose an early intervention for sleep problems to prevent the exacerbation of school non-attendance.

## 1. Introduction

The Ministry of Education, Culture, Sports, Science and Technology (MEXT) of Japan defines “school non-attendance” as absence for 30 or more days in a year due to psychological, emotional, physical, or social factors or circumstances preventing students from attending school, excluding those absent due to illness or economic reasons. According to an MEXT survey [1], the number of children and students classified as absent from school has increased for 11 consecutive years, reaching a record high of 346,482 (3.72%). Therefore, the development of strategies to prevent school non-attendance is an urgent issue.

Protective factors and risk factors contributing to school non-attendance are diverse, with intricately intertwined factors related to school, home, and the child’s own circumstances [2,3]. Protective factors include positive relationships with friends, teachers, and family; learning goals; high level of social functioning; high self-esteem [3]. Poor academic performance, problems in relationships with friends, teachers, and family, and physical or mental health issues are thought to be important risk factors [4]. However, sleep problems, which could be crucial risk factors, are often underestimated, even though numerous reports indicate a relationship between sleep problems and school non-attendance [4,5]. An MEXT survey showed that the percentage of non-attendance students whose parents consulted about irregular sleep–wake rhythms reached 24.5% and 22.1% in junior high and elementary schools, respectively [1]. Furthermore, the Nippon Foundation survey on junior high students showing a tendency toward non-attendance (even if days of absence were fewer than 30, students spent significant time outside their classroom at school or attended school daily while feeling reluctant to do so) found that “feeling tired” and “unable to wake up in the morning” ranked as the top two reasons for not wanting to attend school [6]. Additionally, among junior high students absent for 30 days or more per year, over 50% cited “unable to wake up in the morning” as the reason. Furthermore, our study analyzing factors contributing to school non-attendance revealed that among students experiencing non-attendance, the odds ratio (OR) for “sleep rhythm problems such as difficulty waking up in the morning or difficulty falling asleep at night” was as high as 4.13 compared to those regularly attending school [7]. Another Japanese survey [8] found that within the past month, 10.9% of male and 7.7% of female junior high school students were late because they were unable to get up, while 2.9% of male and 2.0% of female students were absent.

While many students cite sleep problems as a reason for being unable to attend school, the relationship between sleep problems and school non-attendance is suggested to be bidirectional [9]. Sleep problems, such as sleep deprivation and irregular sleep patterns, are reportedly associated with daytime sleepiness [10], mental health deterioration [11,12], and declining academic performance [13,14]. These are all considered risk factors for non-attendance in school. Conversely, students who do not attend school may experience sleep problems like delayed sleep phase due to the lack of a regular daily routine such as school attendance and reduced exposure to morning light.

Various reports have indicated that many school-aged children worldwide also report difficulty waking up, such as 43% of 9- to 14-year-olds in the Netherlands [15], 38.8% of 4th graders and 57.7% of 8th graders in Sweden [16], and 66.9% of 13- to 19-year-olds in Italy [17]. Therefore, the link between difficulty waking up and school non-attendance is not unique to Japan.

The reasons for difficulty in waking up among elementary and junior high school students are thought to be diverse, including sleep deprivation, circadian rhythm disorders, other sleep disorders, and physical or mental illnesses. Sleep deprivation is particularly prevalent in Japan. The American Academy of Sleep Medicine recommends 9–12 h of sleep for healthy children aged 6–12 years and 8–10 h for those aged 13–18 years [18]. However, Japanese elementary and junior high school students’ sleep duration is reported as follows: the average weekday sleep time for elementary school boys is 8.9 h and for girls, 8.8 h [19]; the average sleep time for first-year junior high school boys is 7.9 h and for girls, 7.5 h [20]. This finding indicates that many elementary and junior high school students do not get the recommended amount of sleep. Consequently, on non-school days, they delay waking up to repay their sleep debt. This creates a mismatch in the midpoint of sleep schedules between weekends and weekdays, referred to as social jet lag [21]. Social jet lag is associated with depression [22], anxiety [23], irritable moods, fatigue, and poor academic performance [24] among adolescents. Social jet lag is problematic in itself, but if uncorrected and sleep debt accumulates, difficulty waking up becomes pronounced, potentially progressing to a delayed sleep–wake phase disorder. Once a delayed sleep–wake phase disorder develops, waking up at a time suitable for school attendance becomes increasingly difficult, possibly contributing to being late or not attending school.

To date, no large-scale study has examined the association between sleep regularity and school non-attendance among Japanese elementary and junior high school students. The primary objectives of the current study were to clarify sleep regularity among these students, to elucidate the relationship between sleep irregularity and school attendance status, to identify the characteristics of students with irregular sleep patterns, and to explore intervention methods based on sleep regularity to prevent school non-attendance.

## 2. Materials and Methods

### 2.1. Survey

This study was conducted as part of a project entitled “Research on Factors Related to School Non-Attendance Problems,” commissioned by the MEXT of Japan. This project aimed to identify the factors associated with school non-attendance based on the perspectives of teachers, students, and parents through administered surveys, specifically addressing the gap in the viewpoints of teachers, children, and parents.

The survey was conducted in July 2023. The teacher questionnaire included 63 items concerning each student that the teacher was in charge of during the 2022 academic year, covering topics such as the student’s attendance status (as reported in the national survey), primary contributing factors for non-attendance (if applicable), concerns and complaints that the teacher was aware of during the year, and family background. Two versions of the student questionnaire were administered. One version was designed for students who were not classified as non-attending, that is, those who reported fewer than 30 days of absence during the school year. This version contained 123 items regarding attendance status in 2022, distressing and positive experiences at school and home, personal strengths (protective factors), attendance status in 2023, current difficulties and positive experiences, sleep regularity, diet, and depressive or anxiety symptoms. The non-attendance version included the same items and additional questions about the period when attending school became difficult, learning and daily life at home during that time, whether distressing experiences had been resolved, factors that facilitated improvement, and sources of consultation, for a total of 175 items.

Two versions of the parental questionnaire existed. The attendance version contained 54 items on the child’s attendance status in 2022 and 2023, distressing experiences at school or at home, the child’s strengths, disabilities, or illnesses, and the family’s economic situation. The non-attendance version also asked about the child’s learning and daily life at home during the period of school non-attendance, parents’ perceptions of school responses, and the use of community resources, totaling 124 items.

### 2.2. Participants

The survey was conducted in collaboration with the boards of education from four municipalities, one from each of the following regions: Kanto, Kansai, Chugoku, and Kyushu in Japan. In total, 177 schools participated in the survey, including 91 elementary, 51 junior, and 36 high schools. The participants were students in grades 3–10 (corresponding to the 3rd year of elementary school through the 1st year of high school in Japan) during the 2022 academic year, along with their parents/guardians and homeroom teachers. However, 9th-grade students in the 2022 academic year were excluded from this survey because they advanced to various high schools the following year, making follow-up data collection difficult.

Responses were obtained from homeroom teachers for 25,257 students (response rate: 98.8%). Student questionnaires were completed by 19,005 students (response rate: 75.2%) and parent questionnaires by 12,140 parents or guardians (response rate: 48.1%).

The attendance version was completed by 18,310 students (96.3% of total respondents) and 11,791 parents (97.1%). The non-attendance version was completed by 3.7% of students (*n* = 695) and 2.9% of parents (*n* = 349). This study analyzed data from 19,005 students who completed either of the two student forms.

### 2.3. Questions Used in This Study

The survey items were developed by a team comprising child development specialists, including psychologists and schoolteachers. In this study, we examined only those items common to both the school attendance and non-attendance forms. These included items regarding school attendance status for the 2022 and 2023 academic years, sleep regularity, and items related to sex, grade level, and protective factors against school non-attendance for the 2023 academic year, which were expected to be associated with school attendance and sleep regularity. Students responded to these items.

The items regarding sleep regularity were originally developed from a clinical perspective. As part of a broad survey on school non-attendance, the number of items was limited. Pediatric sleep specialists (IH, MT) selected items that were easy for elementary and junior high school students to recognize and answer, rather than focusing on specific average sleep duration, and that showed strong associations with daytime functioning. The items are as follows (Table 1): (1) “Do you usually get up at about the same time every morning?” (2) “Do you usually go to bed at about the same time every night?” (3) “Is the amount of time you sleep (sleep duration) about the same every day?” For these items, participants responded on a four-point scale: (a) almost the same (difference of less than 1 h), (b) generally the same (difference of less than 2 h), (c) not the same (varies from day to day), or (d) varies depending on whether it is a school day or if there were any plans.

The questions regarding daytime sleepiness were as follows (Table 1): (1) “Are you able to wake up feeling refreshed in the morning?” (2) “Do you ever go back to sleep after waking up (take a second sleep)?” (3) “Do you take naps or doze off during the day?” For these items, responses were given on a three-point scale: (a) yes, (b) no, or (c) varies depending on whether it is a school day or if there are any plans.

Additionally, for school attendance status, the items were rated on a six-point scale. For the 2022 academic year, the number of days absent (excluding absences due to hospitalization, infectious diseases requiring absence, or club activities) was categorized as follows: 0 = 0–15 days, 1 = 15–30 days, 3 = 30–60 days (approximately 1/6 to 1/3 of the school year), 4 = 60–90 days (approximately 1/3 to 1/2 of the school year), 5 = 90–180 days (more than half), and 6 = 180 days or more (almost all). For the 2023 academic year, the number of days attended was categorized as follows: 0 = have not attended at all, 1 = have hardly attended, 2 = about once a week (or 4 days a month, or about 20%), 3 = about 2–3 days a week (or 8–12 days per month, or about 50%), 4 = about 4 days a week (or about 16 days per month, or about 80%), and 5 = attended almost every day or never missed a day.

For protective factors against school non-attendance, we developed items by referencing previous studies outlining risk factors and protective factors related to school attendance problems [2]. The items included yes/no responses for factors such as performance in school life and extracurricular activities, places to belong outside of school, and relationships with schoolteachers, peers, and family in the 2023 academic year, and a five-point scale ranging from 1 (poor) to 5 (very good) for self-reported academic performance (shown in Table 2).

### 2.4. Statistical Analysis

A latent class analysis (LCA) was conducted based on the responses to three survey items regarding sleep regularity and three survey items regarding daytime sleepiness in the 2023 academic year (Table 1). For LCA, 67 individuals who answered two or fewer of the six questions were excluded, resulting in a sample size of 18,938. Latent class models were run from a one-class solution to solutions with an increasing number of classes. To compare models with different numbers of classes and determine the optimal model, we employed several fit indices [25]: the smallest Akaike’s information criterion (AIC), Bayesian information criterion (BIC), sample size-adjusted BIC (ABIC), adjusted Lo–Mendell–Rubin likelihood ratio test (adjusted LMR-LRT), bootstrap likelihood ratio test (BLRT), and entropy. Additionally, the number of classes was determined by integrating other considerations, including theoretical justifications and interpretability.

Chi-square tests were performed to examine differences in characteristics among the identified latent classes based on students’ self-reported responses regarding gender, grade level, attendance status in the 2022 and 2023 academic years, and protective factors in the 2023 academic year. For the 2022 academic year, attendance was defined as less than 30 days absent, and non-attendance as 30 or more days absent. For the 2023 academic year, attendance was defined as attending school 4 days or more per week, and non-attendance as attending fewer than 4 days per week. As academic performance was assessed on a five-point scale, it was treated as a continuous variable, and differences among classes were tested using analysis of variance.

Logistic regression analysis was conducted to explore the factors characterizing the identified latent classes, including school attendance status in the 2022 and 2023 academic years and protective factors against school non-attendance. Many cases were identified in which the child’s response indicated “180 or more days absent” in the 2022 academic year, whereas their parent’s response indicated “0–15 days absent,” and the teacher’s response indicated “not school non-attendance in the 2022 academic year.” These cases were excluded from the analysis because they were clearly not school non-attendance, as evidenced by comments like “I didn’t take any days off in the first place” in the free-response section.

These analyses were performed using Mplus (https://www.statmodel.com/; accessed on 1 December 2025) for the LCA and Stata version 19 (Stata Corp., College Station, TX, USA) for the logistic regression.

For all statistical analyses, the significance level was set at *p* < 0.05.

## 3. Results

### 3.1. Description of the Latent Classes

Table 3 provides an overview of different LCA solutions. The AIC, BIC, and ABIC values continued to decrease up to the five-class solution level, and the adjusted LMR-LRT and BLRT results were significant. Entropy exceeded 0.8 for all class solutions; however, estimation errors in the five-class solution prevented sufficient convergence. Therefore, a four-class solution was selected as the best fit for the current sample in LCA.

**Figure 1:**
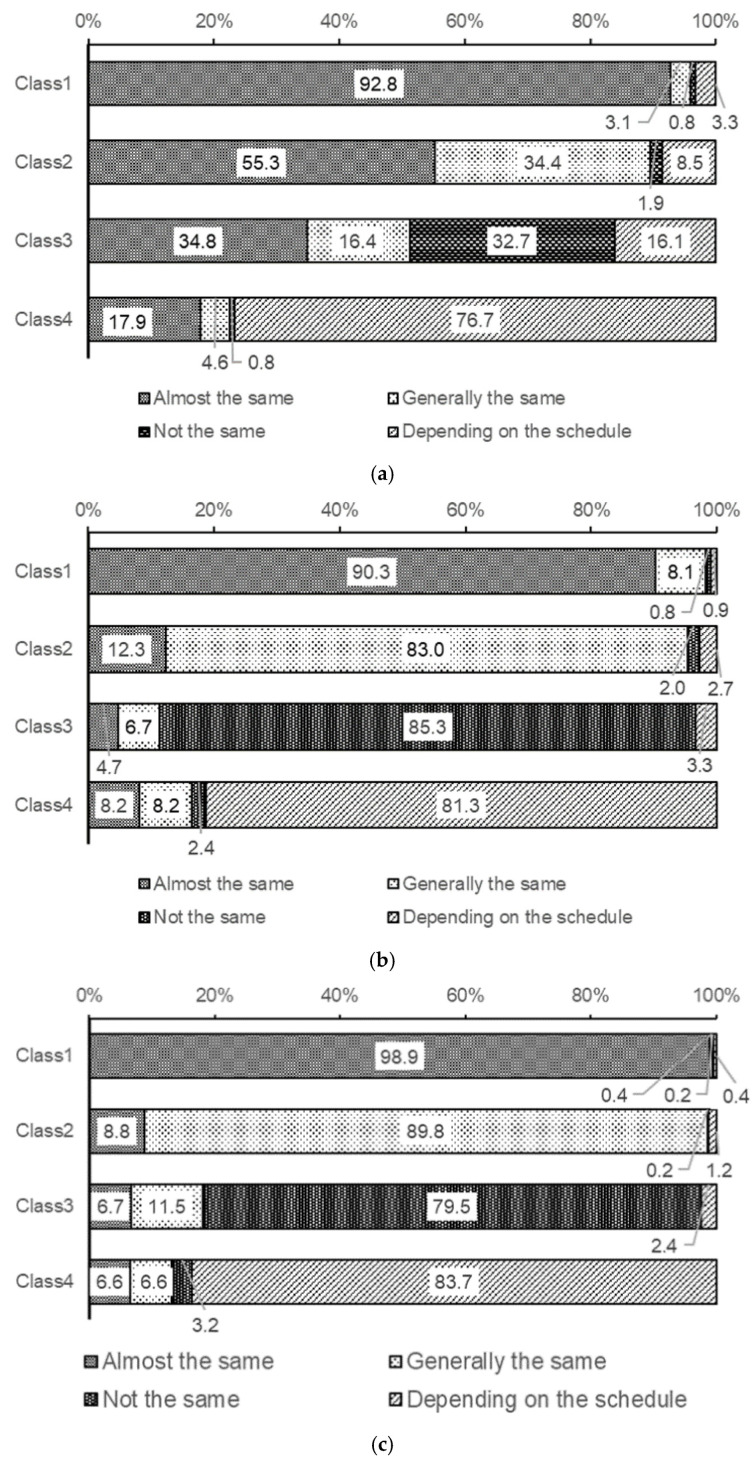
Differences in sleep regularity between the 4 latent classes. (**a**) Regularity of wake-up time. (**b**) Regularity of bedtime. (**c**) Regularity of sleep duration. These figures show the percentage of the answers for the following questions by the students. (**a**) Do you usually get up at about the same time every morning? (**b**) Do you usually go to bed at about the same time every night? (**c**) Is the amount of time you sleep (sleep duration) about the same every day?

Figure 1 shows responses to sleep irregularities across the four classes. In Class 1, 92.8%, 90.3%, and 98.9% of the participants reported “Almost the same” wake-up time, bedtime, and sleep duration, respectively. In Class 2, “Generally the same” was chosen by 34.4%, 83.0%, and 89.8% of participants for wake-up time, bedtime, and sleep duration, respectively. In Class 3, “Not the same” was reported by 32.7%, 85.3%, and 79.5% of participants for wake-up time, bedtime, and sleep duration, respectively. In Class 4, “Depending on the schedule” was chosen by 76.7%, 81.3%, and 83.7% of participants for wake-up time, bedtime, and sleep duration, respectively.

**Figure 2:**
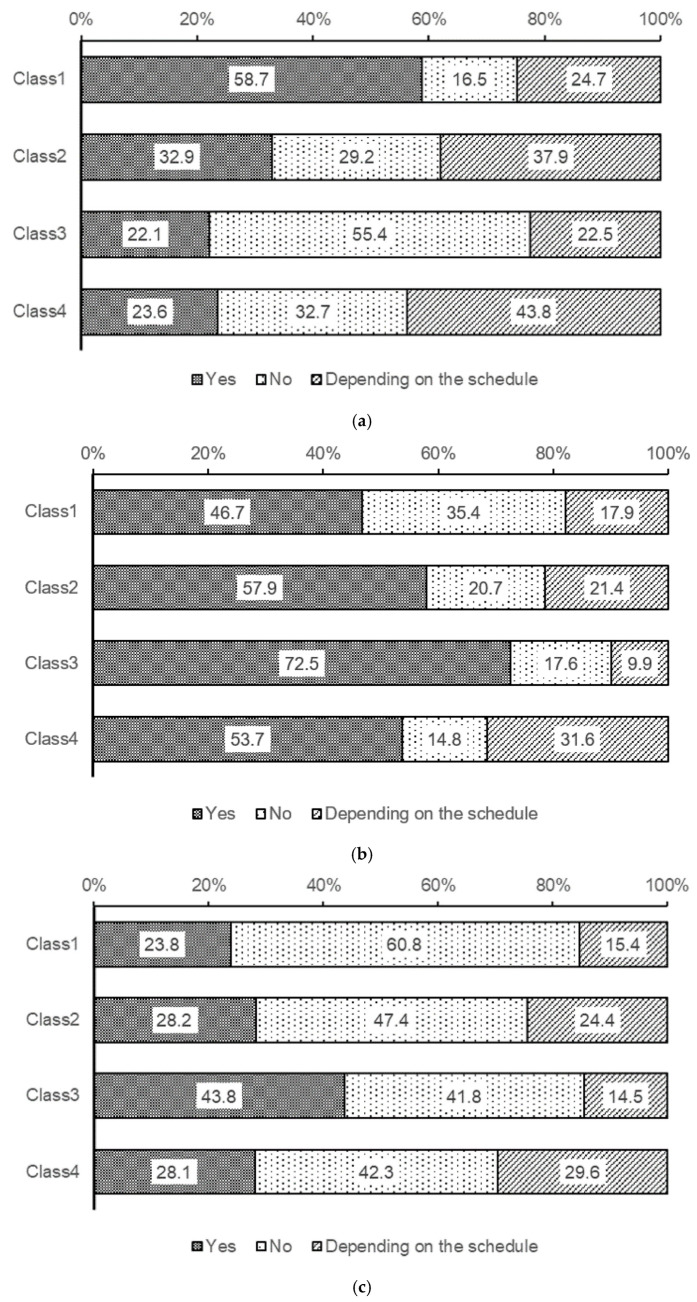
Differences in daytime sleepiness between the 4 latent classes. Figures present the percentage of the answers for the following questions by the students. (**a**) Are you able to wake up feeling refreshed in the morning? (**b**) Do you ever go back to sleep after waking up (take a second sleep)? (**c**) Do you take naps or doze off during the day?

Responses regarding daytime sleepiness across the four classes are shown in Figure 2. In Class 1, 58.7% of the students woke up feeling refreshed, whereas in Class 3, 55.4% did not wake up feeling refreshed. Furthermore, while 46.7% of the students in Class 1 went back to sleep after waking up, 72.5% of the students in Class 3 did. Napping or dozing off during the day was observed in 23.8% of Class 1 students compared to 43.8% of Class 3 students.

Therefore, it was suggested that these four classes reflected groups of students with regular sleep patterns (Class 1; *n* = 9937, 52.5% of the sample), somewhat irregular sleep patterns (Class 2; *n* = 4261, 22.5% of the sample), irregular sleep patterns (Class 3; *n* = 2072, 10.9% of the sample), and schedule-dependent sleep patterns (Class 4; *n* = 2668, 14.1% of the sample).

### 3.2. Comparison of the Characteristics of the Four Latent Classes

The descriptive statistics for the four classes are shown in Table 4. Regarding gender differences across the four classes, boys classified as Class 1, Class 2, Class 3, and Class 4 were 58.0%, 20.5%, 10.6%, and 10.9%, respectively, whereas girls were 47.3%, 24.6%, 11.0%, and 17.2%, respectively. Regarding the relationship between 2023 school attendance status and the four classes, as defined by sleep regularity in the same 2023 academic year, students who reported that “I go to school almost every day” were Class 1 = 53.9%, Class 2 = 22.6%, Class 3 = 9.6%, and Class 4 = 13.9%. Conversely, students who reported that “I go to school about 2 or 3 days a week” were Class 1 = 33.7%, Class 2 = 19.2%, Class 3 = 33.7%, and Class 4 = 13.3%. Among students who reported “I have not attended school at all this year”, Class 1 = 51.7%, Class 2 = 18.5%, Class 3 = 21.9%, and Class 4 = 8.0% (χ^2^(15) = 427.8, *p* < 0.001, V = 0.09).

Supplementary Table S1 presents the same data as Table 4; however, it presents the percentage of students in each class who corresponding to the responses listed on the left column in the Table. Specifically, for the 2023 school year, 92.9% of Class 1 students attended almost every day, compared to 79.6% in Class 3. Furthermore, while 0.9% of Class 1 students hardly attended, this figure was 2.5% for Class 3.

**Figure 3:**
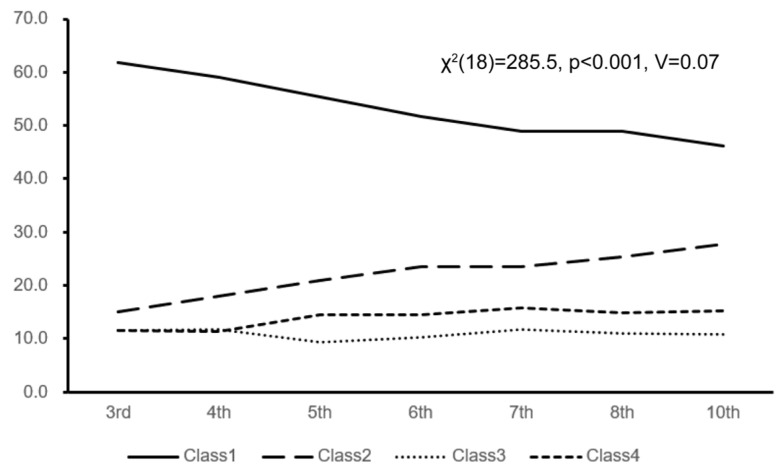
Changes in the proportion of the four latent classes by grade level.

Figure 3 shows the differences in the proportions of the four classes according to the grade level. In the 3rd grade of elementary school, Class 1 accounted for 61.8% of the total, and this proportion decreased with grade progression, reaching 46.2% in 10th grade of high school. The proportions of Class 2 and Class 4 increased as the grade progressed, rising from Class 2 = 15.1% and Class 4 = 11.5% in the 3rd grade to Class 2 = 27.8% and Class 4 = 15.2% in the 10th grade. Class 3 showed little change across grades (χ^2^(18) = 285.5, *p* < 0.001, V = 0.07).

**Figure 4:**
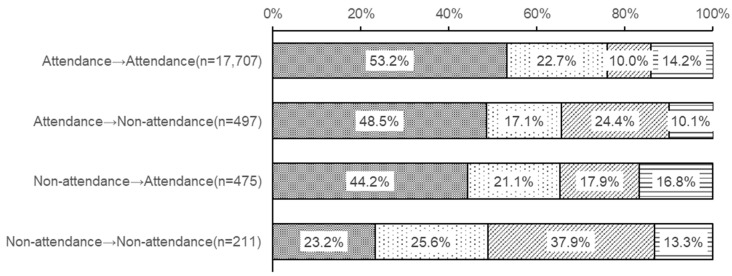
Relationship between changes in school attendance status and the four latency classes. School attendance status was defined as follows: For the 2022 academic year, attendance was defined as fewer than 30 days absent, and non-attendance as 30 or more days absent. For the 2023 academic year, attendance was defined as attending school 4 or more days per week, and non-attendance as attending fewer than 4 days per week. The *X*-axis shows attendance status in 2022 → attendance status in 2023.

Figure 4 shows the relationship between changes in school attendance status between the academic years 2022 and 2023 and the four classes on sleep regularity in 2023. Students maintaining attendance (attendance in both 2022 and 2023) were distributed as follows: Class 1, 53.2%; Class 2, 22.7%; Class 3, 10.0%; Class 4, 14.2%. Students who were defined as attendance in the 2022 academic year but non-attendance in the 2023 academic year were distributed as Class 1 (48.5%), Class 2 (17.1%), Class 3 (24.4%), and Class 4 (10.1%). Students who were non-attendance in the 2022 academic year but attendance in the 2023 academic year were classified as Class 1 (44.2%), Class 2 (21.1%), Class 3 (17.9%), or Class 4 (16.8%). Conversely, students with persistent non-attendance (non-attendance in both 2022 and 2023) were classified as Class 1 (23.2%), Class 2 (25.6%), Class 3 (37.9%), and Class 4 (13.3%).

Table 5 shows differences in protective factors against school non-attendance across the four classes, specifically: presence of a teacher whom the child trusts, presence of close friends, academic performance, proficiency in academic/athletic activities, positive club participation, active involvement in class activities, good communication with others, presence of future goals, positive family relationships, presence of close friends outside school, presence of a place to belong outside school, and presence of other enjoyable activities. Significant differences existed across the four classes for all 13 protective factors. The percentage of students who answered yes to all these questions was highest in Class 1, followed by Class 2, Class 4, and then Class 3. Furthermore, the average of the five-point scale grades was also highest in the following order: Class 1 (mean = 3.52, standard deviation [SD] = 1.15), Class 2 (mean = 3.21, SD = 1.15), Class 4 (mean = 2.84, SD = 1.26), and Class 3 (mean = 3.12, SD = 1.21).

The results of the logistic regression analysis for protective factors across the four groups are shown in Table 6. In terms of demographic variables, girls were observed to have an increased risk of Class 2 (OR = 1.38, 95% confidence interval [CI] = 1.27–1.49, *p* < 0.001), Class 3 (OR = 1.22, 95% CI = 1.09–1.36, *p* < 0.001), and Class 4 (OR = 1.76, 95% CI = 1.60–1.93, *p* < 0.001). School attendance status was significantly associated with Class 3: Attendance in 2022/Non-attendance in 2023 (OR = 1.93, 95% CI = 1.48–2.53, *p* < 0.001), Non-attendance in 2022/Attendance in 2023 (OR = 1.57, 95% CI = 1.18–2.10, *p* < 0.005), and Non-attendance in 2022/Non-attendance in 2023 (OR = 3.12, 95% CI [2.04–4.79], *p* < 0.001). The status of Non-attendance in 2022/Non-attendance in 2023 was also significantly associated with Class 2 (OR = 1.93, 95% CI = 1.24–3.00, *p* < 0.005).

Protective factors significantly and negatively associated with sleep irregularity in all three classes (Class 2, 3, and 4) were: “There is at least one teacher who understands me,” “Academic grades,” “I am good at exercising,” “My communication with others is going well,” “At home, I get along well with my family,” and “There are places where I can have fun outside of school.” “Club activities are going well” was negatively associated with sleep irregularity in Class 3 and 4. “I have a career I want to pursue and a path I hope to take in the future” was negatively associated with sleep irregularity in Classes 2 and 3. “I have close friends outside of school” was negatively associated with sleep irregularity only in Class 2. The factors “I am good at studying” and “Participating in activities such as class meetings, committees, and cultural festivals are going well” were negatively associated only with Class 3. “I have at least one close friend” and “I have other skills and/or things I enjoy” were not significantly associated with any class regarding sleep irregularity. Among the associated factors, the lowest OR, 0.35, was found for “At home, I get along well with my family” (Class 1 vs. Class 3).

## 4. Discussion

### 4.1. Sleep Regularity and Underlying Sleep Problems

Among the causes of irregular sleep patterns in elementary and junior high school students, the main sleep issues are insufficient sleep and social jet lag. Sleep regularity and sleep deprivation have been suggested to be interdependent [26].

Figure 1 and Figure 2 show that Class 1 appeared to have no sleep problems due to their regular patterns. However, many students reported experiencing daytime sleepiness. This suggests that despite having regular sleep schedules, a significant number of students in Class 1 were chronically sleep-deprived. In Classes 2 and 4, compared to Class 1, a higher proportion reported that their morning wakefulness, tendency to return to sleep, and occurrence of daytime naps varied depending on their schedule. This suggests that these students may exhibit irregular sleep patterns to recover sleep debt on days off due to chronic sleep deprivation. While this study did not investigate bedtime and wake-up times, it is presumed that some of these students may be experiencing social jet lag due to sleep deprivation, which is characterized by strong daytime sleepiness and somewhat irregular sleep patterns. Furthermore, Class 3 exhibited the most irregular sleep patterns, with students reporting difficulty in waking up refreshed, frequent napping, and high rates of daytime sleepiness, indicating the most significant sleep problems.

Figure 3 shows that the proportion of students with regular sleep patterns (Class 1) decreased with grade progression, whereas the proportions of students with somewhat irregular sleep patterns (Class 2) and schedule-dependent sleep patterns (Class 4) increased. The primary cause of these changes is a biological shift in the sleep–wake rhythm during adolescence. During adolescence, nocturnal melatonin secretion is delayed, and the sleep phase physiologically shifts backward, leading to a night-type shift of up to approximately 2 h [27]. Furthermore, sleep deprivation is exacerbated by the use of digital devices and nighttime gaming [19,28,29]. Kohyama et al. reported that excessive after-school activities might contribute to sleep deprivation among Japanese students [30]. Meanwhile, on school days, students must wake up at the same time as school starts, leading to a continuous reduction in sleep duration.

These findings clearly indicate that sleep problems among Japanese elementary and junior high school students, particularly sleep deprivation and irregular sleep patterns, worsen with grade level, suggesting the need for early intervention.

### 4.2. Sleep Regularity and School Attendance

To our knowledge, no reports describe the relationship between the rate of non-attendance days and irregular sleep patterns among elementary and junior high school students. With regard to attendance in the 2023 academic year, students attending school 2–3 days per week showed more irregular sleep habits compared to those not attending at all (shown in Table 4). These students may experience irregular sleep patterns due to alternating between school-on days, when they force themselves to wake up, and school-off days, when they do not or cannot wake up. Furthermore, it is possible that the sleep patterns of students with complete absence may have stabilized within the timeframe corresponding to delayed sleep–wake phase disorder, potentially increasing the proportion reporting regular sleep.

Figure 4 shows that students who were non-attending in 2022 but were able to attend school in 2023 were more likely to have regular sleep patterns compared to students who remained non-attending. Conversely, they were more likely to have irregular sleep patterns than students who had consistently attended school. This suggests that the non-attendance state is related to sleep irregularity, and even when students begin attending school again, sleep regularity remains unstable.

Additionally, the proportion of students exhibiting irregular sleep patterns, which is probably associated with sleep deprivation, increases with each grade level (shown in Figure 3). These lines of evidence suggest that intervening in sleep patterns at an early age and stage, when difficulty waking up begins to be noticed and sleep is somewhat irregular, with probable tendencies toward non-attendance beginning to emerge, has the possibility of preventing these tendencies from becoming entrenched in some cases. Furthermore, supporting students who have resumed school attendance by helping them maintain sufficient sleep duration and a regular sleep rhythm during periods of sleep instability may facilitate sustained school attendance.

### 4.3. Relationship Between Sleep Regularity and Protective Factors

Table 5 shows that students with irregular sleep patterns had a higher risk of school non-attendance and lower protective factors against school non-attendance, including relationships with teachers and family, communication skills, academic performance, athletic proficiency, and the presence of a place to belong outside school. Interestingly, students with schedule-dependent sleep patterns (Class 4) showed low protective factors similar to those with irregular sleep patterns.

These findings suggest that low protective factors may increase susceptibility to school non-attendance, and that irregular sleep patterns become more pronounced in situations of school non-attendance. Alternatively, low protective factors may increase susceptibility to irregular sleep patterns, which in turn increases susceptibility to school non-attendance. Both of these mechanisms may be involved in this process.

In this study, students with various degrees of irregular sleep patterns showed problems in family relationships (Table 5). Although the school-aged children surveyed in this study required family cooperation to maintain regular sleep, problems in family relationships might have made it difficult to obtain this cooperation.

Students with schedule-dependent sleep patterns tended to have irregular wake-ups and bedtimes on days without plans, yet many still attended school. We predicted that students who showed a willingness to attend school would adapt better to school life than those with irregular sleep. However, the results suggest that they may have similar maladaptive problems, yet their attendance patterns make it difficult to identify these problems.

Compared to the psychosocial background of school non-attendance, which is difficult to identify early within limited timeframes at school, sleep regularity, an aspect easier to assess with students and their families, was considered a potential barometer for children experiencing difficulties.

### 4.4. The Necessity of Sleep Intervention

From a sleep perspective, preventing school non-attendance requires interventions for sleep deprivation and social jet lag. Japanese children have had short sleep durations since infancy [31], suggesting the need for sleep education for children, families, and support providers from an early age. In Japan, sleep education initiatives where children learn about appropriate sleep habits at school have been reported to be effective in reducing school non-attendance [32].

As sleep and school non-attendance are considered bidirectional issues, intervening in school non-attendance may also improve sleep problems.

Furthermore, students with school non-attendance exhibited noticeable sleep irregularities, and even after resuming attendance, their sleep regularity remained fragile. To intervene effectively, it is useful not only for families but also for all potential supporters of children with school non-attendance, such as teachers and school counselors, to have accurate knowledge about sleep.

## 5. Conclusions

Students exhibiting irregular sleep habits were more common among those experiencing non-attendance, and the proportion of students with irregular sleep habits increased with higher levels of non-attendance.

Students with irregular sleep patterns have fewer protective factors against school non-attendance.

We conclude that sleep education for children and their families may help prevent school non-attendance or mitigate prolonged sleep irregularity.

This study had some limitations:

1. This study assessed indicators of sleep regularity and sleepiness but did not include bedtime, wake-up time, or sleep duration. Therefore, we could not directly evaluate the presence of sleep deprivation, social jet lag and circadian rhythm disorders in the children.
2. Sleep regularity was assessed only for the 2023 academic year, preventing evaluation of temporal changes related to school non-attendance.
3. This study relied entirely on self-reported responses from students and did not utilize sleep diaries or objective actigraphy devices.
4. This study was not longitudinal, which prevented establishing a temporal relationship between sleep irregularity and school non-attendance. The relationship is probably bidirectional; some students may develop school non-attendance due to sleep problems, while others may develop sleep problems due to school non-attendance.

## Figures and Tables

**Table 1:** Indicator variables used for latent class analysis.

Questions	Answers			
	0	1	2	3
Do you usually get up at about the same time every morning?	Not the same (varies from day to day).	Generally the same (difference of <2 h).	Almost the same (difference of <1 h).	It varies depending on whether it’s a school day or if there are any plans.
Do you usually go to bed at about the same time every night?	Not the same (varies from day to day).	Generally the same (difference of <2 h).	Almost the same (difference of <1 h).	It varies depending on whether it’s a school day or if there are any plans.
Is the amount of time you sleep (sleep duration) about the same every day?	Not the same (varies from day to day).	Generally the same (difference of <2 h).	Almost the same (difference of <1 h).	It varies depending on whether it’s a school day or if there are any plans.
Are you able to wake up feeling refreshed in the morning?	No		Yes	It varies depending on whether it’s a school day or if there are any plans.
Do you ever go back to sleep after waking up (take a second sleep)?	No		Yes	It varies depending on whether it’s a school day or if there are any plans.
Do you take naps or doze off during the day?	No		Yes	It varies depending on whether it’s a school day or if there are any plans.

**Table 2:** Questionnaire about protective factors against school non-attendance. Students answered these questions as yes or no (excluding the five-point academic performance scale).

Protective Factors
There is at least one teacher who understands me.
I have at least one close friend.
Academic grades: 5-point scale ranging from 1 (poor) to 5 (very good)
1. I am good at studying.
2. I am good at exercising.
3. Club activities are going well.
4. Participating in activities such as class meetings, committees, and cultural festivals is going well.
5. My communication with others is going well.
6. I have a career I want to pursue and a path I hope to take in the future.
7. At home, I get along well with my family.
8. I have close friends outside of school.
9. There are places where I can have fun outside of school.
10. Besides the above 1–9, I have other skills and/or things I enjoy.

**Table 3:** Overview of the solutions from the LCA.

Class Solutions	AIC	BIC	ABIC	Entropy	Adjusted LMR-LRT *p*-Value	BLRT *p*-Value
2-class	220,319.738	220,563.055	220,464.539	0.863	<0.001	<0.001
3-class	212,989.681	213,358.580	213,209.217	0.881	<0.001	<0.001
4-class	208,029.293	208,523.776	208,323.565	0.882	<0.001	<0.001
5-class *	206,280.960	206,901.026	206,649.968	0.820	<0.001	<0.001

Abbreviation: AIC, Akaike’s information criterion; BIC, Bayesian information criterion; ABIC, sample size adjusted BIC; LMR-LRT, Lo–Mendell–Rubin likelihood ratio test; BLRT, bootstrap likelihood ratio test. * estimation error occurred.

**Table 4:** Differences in the characteristics between the four latent classes.

	Class 1 (*n* = 9937, 52.5%)	Class 2 (*n* = 4261, 22.5%)	Class 3 (*n* = 2072, 10.9%)	Class 4 (*n* = 2668, 14.1%)	Differences Between Classes Total Number of People
**Gender**					**Χ^2^(6) = 324.2, *p* < 0.001, V = 0.09**
Boys	5472 (58.0%)	1938 (20.5%)	1000 (10.6%)	1032 (10.9%)	9442 (100%)
Girls	4382 (47.3%)	2277 (24.6%)	1023 (11.0%)	1590 (17.2%)	9272 (100%)
Others	37 (26.8%)	30 (21.7%)	36 (26.1%)	35 (25.4%)	138 (100%)
**Number of school absences in 2022**					**Χ^2^(15) = 296.0, *p* < 0.001, V = 0.07**
0–15 days	9344 (53.7%)	3906 (22.5%)	1738 (10.0%)	2412 (13.9%)	17,400 (100%)
15–30 days	330 (39.5%)	193 (23.1%)	165 (19.8%)	147 (17.6%)	835 (100%)
30–60 days	95 (36.0%)	52 (19.7%)	64 (24.2%)	53 (20.1%)	264 (100%)
60–90 days	28 (26.4%)	30 (28.3%)	29 (27.4%)	19 (17.9%)	106 (100%)
90–180 days	31 (28.4%)	32 (29.4%)	34 (31.2%)	12 (11.0%)	109 (100%)
>180 days	48 (41.4%)	28 (24.1%)	27 (23.3%)	13 (11.2%)	116 (100%)
**Number of school attendance days in 2023**					**Χ^2^(15) = 427.8, *p* < 0.001, V = 0.09**
I haven’t attended school at all this year.	78 (51.7%)	28 (18.5%)	33 (21.9%)	12 (8.0%)	151
I have hardly attended school this year.	87 (45.8%)	34 (17.9%)	51 (26.8%)	18 (9.5%)	190
I go to school about once a week.	39 (34.8%)	28 (25.0%)	31 (27.7%)	14 (12.5%)	112
I go to school about 2 or 3 days a week.	86 (33.7%)	49 (19.2%)	86 (33.7%)	34 (13.3%)	255
I go to school about 4 days a week.	414 (37.7%)	259 (23.6%)	219 (19.9%)	207 (18.8%)	1099
I go to school almost every day, or I haven’t missed a single day.	9207 (53.9%)	3857 (22.6%)	1641 (9.6%)	2378 (13.9%)	17,083

**Table 5:** Differences in the protective factors against school non-attendance between the four latent classes. This table presents the number and percentage of students who answered “yes” to questions about protective factors in each class. For academic grades, it shows the average of the five-point scale ratings for each class.

	Class 1 (*n* = 9937, 52.5%)	Class 2 (*n* = 4261, 22.5%)	Class 3 (*n* = 2072, 10.9%)	Class 4 (*n* = 2668, 14.1%)	Differences Between Classes
**Protective factors against school non-attendance**	Number and percentage of students who answered “Yes” (excluding Academic grades)				
There is at least one teacher who understands me.	7078 (72.9%)	2647 (63.5%)	1124 (55.6%)	1455 (55.8%)	Χ^2^(3) = 438.2, *p* < 0.001, V = 0.15
I have at least one close friend.	9581 (98.8%)	4090 (97.9%)	1929 (95.8%)	2525 (96.7%)	Χ^2^(3) = 100.0, *p* < 0.001, V = 0.07
Academic grades: 5-point scale ranging from 1 (poor) to 5 (very good)	mean = 3.52, SD = 1.15	mean = 3.21, SD = 1.15	mean = 2.84, SD = 1.26	mean = 3.12, SD = 1.21	F(3, 18,587) = 252.1, *p* < 0.001, η^2^ = 0.04
1. I am good at studying.	4421 (44.7%)	1435 (33.8%)	468 (22.7%)	874 (32.9%)	Χ^2^(3) = 454.2, *p* < 0.001, V = 0.16
2. I am good at exercising.	6535 (66.2%)	2315 (54.7%)	1048 (51.0%)	1313 (49.7%)	Χ^2^(3) = 393.3, *p* < 0.001, V = 0.14
3. Club activities are going well.	8237 (83.3%)	3266 (77.2%)	1338 (65.0%)	1934 (73.0%)	Χ^2^(3) = 410.9, *p* < 0.001, V = 0.15
4. Participating in activities such as class meetings, committees, and cultural festivals is going well.	9163 (92.8%)	3785 (89.5%)	1576 (76.9%)	2322 (87.7%)	Χ^2^(3) = 471.1, *p* < 0.001, V = 0.16
5. My communication with others is going well.	9108 (92.3%)	3657 (86.2%)	1580 (76.7%)	2226 (84.1%)	Χ^2^(3) = 477.8, *p* < 0.001, V = 0.16
6. I have a career I want to pursue and a path I hope to take in the future.	7562 (76.5%)	2937 (69.1%)	1329 (64.6%)	1829 (69.0%)	Χ^2^(3) = 182.8, *p* < 0.001, V = 0.10
7. At home, I get along well with my family.	9545 (96.6%)	3970 (93.9%)	1757 (85.3%)	2413 (91.2%)	Χ^2^(3) = 433.3, *p* < 0.001, V = 0.15
8. I have close friends outside of school.	8652 (87.8%)	3537 (83.6%)	1692 (82.3%)	2205 (83.1%)	Χ^2^(3) = 83.6, *p* < 0.001, V = 0.07
9. There are places where I can have fun outside of school.	7949 (80.7%)	3048 (72.0%)	1314 (64.1%)	1827 (69.0%)	Χ^2^(3) = 376.3, *p* < 0.001, V = 0.14
10. Besides the above 1–9, I have other skills and/or things I enjoy.	7689 (78.3%)	3110 (73.5%)	1404 (68.6%)	1925 (73.1%)	Χ^2^(3) = 111.4, *p* < 0.001, V = 0.08

**Table 6:** Odds ratios of the three latent classes relative to Class 1.

	Class 1	Class 2	Class 3	Class 4
		OR (CI)	OR (CI)	OR (CI)
**Grade**	Ref	1.09 **** (1.07, 1.11)	0.93 **** (0.91, 0.95)	1.01 (0.99, 1.04)
**Gender**: Girl	Ref	1.38 **** (1.27, 1.49)	1.22 **** (1.09, 1.36)	1.76 **** (1.60, 1.93)
Others	Ref	1.65 (0.98, 2.77)	2.80 **** (1.65, 4.74)	3.08 **** (1.85, 5.13)
**School attendance status**				
Attendance in 2022→Attendance in 2023		(base)	(base)	(base)
Attendance in 2022→Non-attendance in 2023	Ref	0.86 (0.65, 1.14)	1.93 **** (1.48, 2.53)	0.81 (0.58, 1.13)
Non-attendance in 2022→Attendance in 2023	Ref	0.99 (0.76, 1.29)	1.57 *** (1.18, 2.10)	1.21 (0.91, 1.61)
Non-attendance in 2022→Non-attendance in 2023	Ref	1.93 *** (1.24, 3.00)	3.12 **** (2.04, 4.79)	1.31 (0.77, 2.20)
**Protective factors against school non-attendance**				
There is at least one teacher who understands me.	Ref	0.81 **** (0.74, 0.88)	0.71 **** (0.64, 0.80)	0.61 **** (0.55, 0.67)
I have at least one close friend.	Ref	1.08 (0.79, 1.48)	1.04 (0.74, 1.45)	0.82 (0.59, 1.13)
Academic grades (5-point scale)	Ref	0.91 **** (0.87, 0.95)	0.76 **** (0.72, 0.80)	0.85 **** (0.81, 0.89)
1. I am good at studying.	Ref	0.94 (0.86, 1.04)	0.64 **** (0.56, 0.74)	0.98 (0.88, 1.10)
2. I am good at exercising.	Ref	0.85 **** (0.79, 0.93)	0.85 **** (0.76, 0.95)	0.76 **** (0.69, 0.84)
3. Club activities are going well.	Ref	0.95 (0.86, 1.06)	0.70 **** (0.62, 0.80)	0.80 **** (0.71, 0.90)
4. Participating in activities such as class meetings, committees, and cultural festivals is going well.	Ref	0.91 (0.78, 1.05)	0.63 **** (0.54, 0.74)	0.90 (0.76, 1.07)
5. My communication with others is going well.	Ref	0.74 **** (0.65, 0.85)	0.64 **** (0.54, 0.75)	0.82 * (0.70, 0.95)
6. I have a career I want to pursue and a path I hope to take in the future.	Ref	0.83 **** (0.76, 0.91)	0.88 * (0.79, 0.99)	0.89 (0.80, 0.99)
7. At home, I get along well with my family.	Ref	0.67 **** (0.56, 0.81)	0.35 **** (0.29, 0.42)	0.54 **** (0.45, 0.66)
8. I have close friends outside of school.	Ref	0.89 * (0.79, 0.99)	1.14 (0.98, 1.33)	1.00 (0.87, 1.14)
9. There are places where I can have fun outside of school.	Ref	0.89 * (0.81, 0.98)	0.76 **** (0.67, 0.87)	0.80 **** (0.71, 0.90)
10. Besides the above 1–9, I have other skills and/or things I enjoy.	Ref	0.98 (0.90, 1.08)	1.05 (0.93, 1.19)	1.07 (0.96, 1.20)

The data with statistical significance are shown in bold. * *p* < 0.05, *** *p* < 0.005, **** *p* < 0.001. OR, odds ratio; CI, confidence interval; Ref, reference category. School attendance status was defined as follows: For the 2022 academic year, attendance was defined as fewer than 30 days absent, and non-attendance as 30 or more days absent. For the 2023 academic year, attendance was defined as attending school 4 or more days per week, and non-attendance as attending fewer than 4 days per week.

## Data Availability

The data presented in this study are available on request from the corresponding author due to restrictions (privacy and ethical reasons).

## Supplementary Materials

The following supporting information can be downloaded at https://www.mdpi.com/article/10.3390/children13010080/s1, Table S1: Differences in characteristics between the four latent classes (alternative version).

## Abbreviations

The following abbreviations are used in this manuscript:

MEXT	Ministry of Education, Culture, Sports, Science and Technology (Japan)
LCA	Latent Class Analysis
AIC	Akaike’s Information Criterion
BIC	Bayesian Information Criterion
ABIC	Sample Size-Adjusted Bayesian Information Criterion
LMR-LRT	Lo–Mendell–Rubin Likelihood Ratio Test (Adjusted)
BLRT	Bootstrap Likelihood Ratio Test
