# Supplementary material for: Relationship Between Sleep Irregularity and School Non-Attendance Among Japanese Elementary and Junior High School Students
Source: Children (Basel). 2026 Jan 4;13(1):80. doi: 10.3390/children13010080 (PMC12840487; doi:10.3390/children13010080)
Supplement: Supplementary file 1 [file children-13-00080-s001.zip › children-4027273-supplementary.pdf]

Table S1: Differences in the characteristics between the four latent classes (another version).

Data presented in this table is the same data as Table 4; however, it shows the percentage of students in each class who gave the responses listed on the left.

	Class 1 (n=9937, 52.5%)	Class 2 (n=4261, 22.5%)	Class 3 (n=2072, 10.9%)	Class 4 (n=2668, 14.1%)	Differences between classes Total number of people
Gender					$X^2(6)=324.2$, $p<0.001$, V=0.09
Boys	5472 (55.3%)	1938 (45.7%)	1000 (48.6%)	1032 (38.8%)	9,442 (50.1%)
Girls	4382 (44.3%)	2277 (53.6%)	1023 (49.7%)	1590 (59.8%)	9,272 (49.2%)
Others	37 (0.4%)	30 (0.7%)	36 (1.8%)	35 (1.3%)	138 (0.7%)
Number of school absences in 2022					$X^2(15)=296.0$, $p<0.001$, V=0.07
0–15 days	9344 (94.6%)	3906 (92.1%)	1738 (84.5%)	2412 (90.8%)	17,400 (92.4%)
15–30 days	330 (3.3%)	193 (4.6%)	165 (8.0%)	147 (5.5%)	835 (4.4%)
30–60 days	95 (1.0%)	52 (1.2%)	64 (3.1%)	53 (2.0%)	264 (1.4%)
60–90 days	28 (0.3%)	30 (0.7%)	29 (1.4%)	19 (0.7%)	106 (0.6%)
90–180 days	31 (0.3%)	32 (0.8%)	34 (1.7%)	12 (0.5%)	109 (0.6%)
>180 days	48 (0.5%)	28 (0.7%)	27 (1.3%)	13 (0.5%)	116 (0.6%)
Number of school attendance days in 2023					$X^2(15)=427.8$, $p<0.001$, V=0.09
I haven't attended school at all this year.	78 (0.8%)	28 (0.7%)	33 (1.6%)	12 (0.5%)	151 (0.8%)
I have hardly attended school this year.	87 (0.9%)	34 (0.8%)	51 (2.5%)	18 (0.7%)	190 (1.0%)
I go to school about once a week.	39 (0.4%)	28 (0.7%)	31 (1.5%)	14 (0.5%)	112 (0.6%)
I go to school about 2 or 3 days a week.	86 (0.9%)	49 (1.2%)	86 (4.2%)	34 (1.3%)	255 (1.4%)
I go to school about 4 days a week.	414 (4.2%)	259 (6.1%)	219 (10.6%)	207 (7.8%)	1099 (5.8%)
I go to school almost every day, or I haven't missed a single day.	9207 (92.9%)	3857 (90.7%)	1641 (79.6%)	2378 (89.3%)	17,083 (90.4%)
